# Do food-related capabilities, opportunities and motivations of adolescents mediate the association between socioeconomic position in adolescence and diet quality in early adulthood?

**DOI:** 10.1186/s12966-023-01477-3

**Published:** 2023-06-12

**Authors:** Katherine M. Livingstone, Dana Lee Olstad, Sarah A. McNaughton, Sara Nejatinamini, James Dollman, David Crawford, Anna Timperio

**Affiliations:** 1 Institute for Physical Activity and Nutrition (IPAN), School of Exercise and Nutrition Sciences, Deakin University, Geelong, VIC 3220 Australia; 2 Department of Community Health Sciences, Cumming School of Medicine, University of Calgary, Calgary, Canada; 3 Alliance for Research in Exercise, Nutrition and Activity, University of South Australia, Adelaide, 5000 Australia

**Keywords:** Structural equation modelling, Diet quality, Dietary behaviours, Dietary determinants, Adolescents, Young adults, Early adulthood; longitudinal

## Abstract

**Background:**

Socio-economic position (SEP) in adolescence may influence diet quality over the life course. However, knowledge of whether individual and environmental determinants of diet quality mediate the longitudinal association between SEP and diet quality is limited. This study examined whether and to what extent food-related capabilities, opportunities and motivations of adolescents mediated the longitudinal association between SEP in adolescence and diet quality in early adulthood overall and by sex.

**Methods:**

Longitudinal data (annual surveys) from 774 adolescents (16.9 years at baseline; 76% female) from ProjectADAPT (T1 (baseline), T2, T3) were used. SEP in adolescence (T1) was operationalized as highest level of parental education and area-level disadvantage (based on postcode). The Capabilities, Opportunities and Motivations for Behaviour (COM-B) model was used as a framework to inform the analysis. Determinants in adolescence (T2) included food-related activities and skills (Capability), home availability of fruit and vegetables (Opportunity) and self-efficacy (Motivation). Diet quality in early adulthood (T3) was calculated using a modified version of the Australian Dietary Guidelines Index based on brief dietary questions on intake of foods from eight food groups. Structural equation modelling was used to estimate the mediating effects of adolescents’ COM-B in associations between adolescent SEP and diet quality in early adulthood overall and by sex. Standardized beta coefficients (β) and robust 95% confidence intervals (CI) were generated, adjusted for confounders (T1 age, sex, diet quality, whether still at school, and living at home) and clustering by school.

**Results:**

There was evidence of an indirect effect of area-level disadvantage on diet quality via Opportunity (β: 0.021; 95% CI: 0.003 to 0.038), but limited evidence for parental education (β: 0.018; 95% CI: -0.003 to 0.039). Opportunity mediated 60.9% of the association between area-level disadvantage and diet quality. There was no evidence of an indirect effect via Capability or Motivation for either area-level disadvantage or parental education, or in males and females separately.

**Conclusions:**

Using the COM-B model, the home availability of fruit and vegetables (Opportunity) of adolescents explained a large proportion of the association between area-level disadvantage in adolescence and diet quality in early adulthood. Interventions to address poor diet quality among adolescents with a lower SEP should prioritize environmental determinants of diet quality.

**Supplementary Information:**

The online version contains supplementary material available at 10.1186/s12966-023-01477-3.

## Introduction

Adolescents, broadly defined as individuals aged 12 to 17 years, have some of the poorest diets of all age groups [1, 2]. In particular, high intake of discretionary foods and beverages and low intake of fruit and vegetables contribute to low overall diet quality in Australian adolescents [3]. Poor diet quality in adolescence substantially impacts health across the lifecourse, including increased risk of obesity and psychological distress in adolescence and early adulthood, as well as chronic disease and premature mortality in later life [4]. Moreover, the pathways through which poor diet quality in adolescence impacts health throughout life are likely to be sensitive to dietary and health inequities that track over time [5].

Socio-economic position (SEP) is a predictor of dietary and health inequities, with evidence to suggest low SEP in adolescence predicts low diet quality in later life [5]. Data from Australia and internationally indicate that dietary inequities have persisted over time, [6, 7] and individual-level indicators of SEP, such as low parental education, as well as environmental-level indicators, such as high area-level disadvantage, are among the strongest predictors of low diet quality in adolescence [6, 8]. Thus, investigating the influence of both individual and environmental-level indicators of SEP on diet quality is important to design policy and behaviour change interventions that address barriers to healthy eating experienced by adolescents with a low SEP.

The Capacities, Opportunities and Motivations model of Behaviour (COM-B) is a theoretical framework that can be used to classify determinants of diet quality at multiple levels [9]. This model recognizes that for any behaviour to occur, individuals must have the physical and psychological capability to enact it, the physical and social opportunity to engage in the behaviour, and be motivated to do it [9, 10]. Having skills for food planning, shopping and preparation (i.e., Capabilities), the availability of and access to fruit and vegetables at home (i.e., Opportunity) and self-efficacy to eat healthy regardless of the food environment (i.e., Motivation), are all associated with higher diet quality in adolescence [11–14]. As some of these determinants, such as having food planning skills, track into early adulthood, [15] they may help to maintain better diet quality during the transition from adolescence to early adulthood. These determinants are also likely to be socially patterned; for example, adolescents with a low SEP have been shown to have lower access to fruit and vegetables compared to adolescents with a high SEP [16]. Thus, COM determinants have the potential to mediate associations between SEP and diet quality. Further, the extent of these influences may differ between males and females since adolescent females have been reported to have higher food involvement skills and motivation to eat healthily [14, 17–19].

Application of the COM-B model can advance understanding of whether and to what extent dietary determinants in adolescence help explain, or mediate, the association between SEP in adolescence and diet quality in early adulthood. Although this model has been applied to understand determinants of diet quality in adolescents and young adults, [19] most evidence for how COM mediate the association between SEP and dietary intake has focused on fruit and vegetables only and has been in mid-aged adults [20]. Further, most research in adolescents has investigated individual-level and environmental-level dietary mediators in isolation, which does not allow for quantification of joint-mediating effects. To our knowledge, no prospective research has modelled potential mediators between low SEP and poor diet quality during this unique transitional life stage, and whether these mediating pathways are comparable across indicators of SEP or between males and females. Therefore, this study aimed to examine whether and to what extent food-related capabilities, opportunities and motivations of adolescents mediate the longitudinal association between SEP in adolescence and diet quality in early adulthood overall and by sex.

## Materials and methods

### Study design

The present study used baseline (T1), 1-year (T2) and 2-year (T3) annual survey data from the ProjectADAPT study; a longitudinal study that recruited adolescents in Year 11 (approximately 16–17 years old) and tracked their dietary practices and behaviours into early adulthood (approximately 18–19 years old). Baseline survey data (T1) were collected between August 2013 and July 2015. Participants were followed up approximately one (T2) and two (T3) years later. Surveys were designed to take less than 30 min for participants to complete and included 66 items (eating behaviours, physical activity, sedentary behaviour, social environment, neighbourhood context, life changes and general health). Participants received a AUD$20 voucher from a major department store after completing the T1 survey and after completing the T2 survey. They received a AUD$25 voucher at T3. This study was conducted according to the guidelines laid down in the Declaration of Helsinki and all procedures involving human subjects were approved by Deakin University’s Human Ethics Advisory Group – Health (159_2012) and relevant education authorities. Written informed consent was obtained from parents and written assent was obtained from adolescents. The reporting of this manuscript was guided by the Strengthening the Reporting of Observational Studies in Epidemiology—Nutritional Epidemiology (STROBE-nut) statement (Supplemental Table 1) [21].

### Participants

Participants were recruited through secondary schools and online social media advertising, as previously described [22]. Schools with at least 50 students enrolled in Year 11 (second-last year of secondary school in Australia) were approached from each tertile of area-level socio-economic disadvantage within (i) major cities and (ii) regional/remote areas of Victoria, based on postcode. Area-level disadvantage was based on the Australian Bureau of Statistics’ 2011 Index of Relative Socio-Economic Advantage and Disadvantage [23]. Overall, 232 schools were invited to participate and 47 consented (29% response rate). Participating schools distributed information about the study to Year 11 students from August 2013 to September 2014. Consent was received from 411 students (5% of recruitment packs distributed) and 382 students completed the survey. To increase the sample size, paid advertising was placed on an online social networking site (Facebook) on two occasions (September to November 2014; April to May 2015). The advertisement targeted adolescents aged 16–17 years living in Victoria, Australia. The advertisement led individuals to a study website where they were screened and could register to receive detailed information about the study. Of the 2770 registrations of interest, consent was received from 655 students, and 640 students completed the baseline survey. In total, considering all those recruited from schools and via social media, 1076 consent forms were received, and 1022 participants completed baseline surveys. Participants recruited in 2013 completed a telephone interview. From 2014, participants could complete surveys online or via telephone. Telephone interviews were completed by 76 participants in total.

### SEP in adolescence (exposures)

Highest parental education and area-level disadvantage at baseline (T1) were selected as indicators of individual-level and environment-level SEP, respectively. Highest parental education was reported by adolescents for their mother/female carer and father/male carer, respectively as: (i) never attended school; (ii) primary school; (iii) some high school; (iv) completed high school; (v) technical or trade school certificate or an apprenticeship; (vi) university or tertiary qualification; and (vii) not applicable/no mother/father carer. For this analysis, the response options for mothers and fathers were combined to reflect highest parental education, which is consistent with literature suggesting parental education is an important independent determinant of diet quality in adolescence [24] and the influence of maternal and paternal education on the child are more comparable during adolescence than during infancy [25]. Parental education was collapsed into three categories: low (some high school or less); medium (completed high school, or technical or trades school certificate or an apprenticeship); and high (university or tertiary qualification) [22]. The Australian Bureau of Statistics’ 2011 Index of Relative Socio-Economic Advantage and Disadvantage (T1) was used as an area-level indicator of SEP in adolescence. This index summarizes information about the economic and social conditions of people and households within an area and was obtained for the postcode in which participants reported living most of the time [23]. Tertiles for area-level disadvantage were used, where a lower score (first tertile) indicated that participants were living in an area with a relatively high prevalence of disadvantage and a low prevalence of advantage.

### Determinants of diet quality in adolescence (mediators)

Dietary determinants were selected at T2 to represent mediators between T1 (exposure) and T3 (outcomes). These determinants were classified as Capabilities, Opportunities and Motivation consistent with the COM-B theoretical domains (Table 1) [9, 26]. Capability is defined as “an individual’s psychological and physical capacity to engage in the activity concerned” [9]. In this study, determinants classified as Capability included five food-related activities and skills. Participants indicated how many times in the past month they: (1) shopped for food, or helped to shop for food, for their household; (2) planned ahead what they would eat at meals at home; (3) made a grocery shopping list for their household; (4) prepared a meal for their household on their own; and (5) helped to prepare a meal for their household. Response options ranged from “Never/rarely” to “Every-day” [15]. These food-related activities and skills have previously been identified as determinants of diet quality in adolescents [27]. Opportunity is defined as “all the factors that lie outside the individual that make behaviour possible or prompt it”, including physical and social factors [9]. In this study, determinants classified as Opportunity related to home availability of fruits and vegetables. Survey items requested how often: (1) fruit and (2) vegetables were available at home, with response options ranging from “Never” to “Always” [28, 29]. Having a large selection of fresh fruit and vegetables in the neighborhood (a statement with response options ranging from “Strongly agree” to “Strongly disagree”) was considered for inclusion in the latent variable for Opportunity (as described later) [30]. These items represented environmental-level dietary determinants in adolescence. Motivation is defined as all the “brain processes that energise and direct behaviour, not just goals and conscious decision-making” [9]. In this study, self-efficacy was classified as Motivation. Participants were asked how confident they felt that they could eat healthy foods when they were: (1) at the shops; (2) hungry after school or work; (3) with their friends; (4) feeling down, bored or stressed; (5) eating out; and (6) alone. Response options ranged from “Not at all” to “Extremely confident” [31].

**Table 1:** Variables from the ProjectADAPT study classified as determinants using Michie’s Behavour Change Wheel Capability, Opportunity, Motivation and Behaviour (COM-B) model

Source of behaviour (COM-B)	Theoretical domain framework (COM-B)	Determinant	Survey items	Response options
Capability – Physical	Physical skills	Food-related activities and skills (5 questionnaire items)	How many times in the past month did you (1) shop for food, or help shop for food, for your household; (2) plan ahead what you would eat at meals at home; (3) make a grocery shopping list for your household; (4) prepare a meal for your household on your own; (5) help to prepare a meal for your household.	Never/rarely; less than once/week; once/week; about 2–3 times/week; about 4–6 times/week; every day
Opportunity - Physical	Environmental context and resources	Availability of healthy food (2 questionnaire items)	How often (1) is fruit available in your home; (2) are vegetables available in your home	Sometimes; usually; always.
Motivation - Reflective	Beliefs about capabilities	Self-efficacy (6 questionnaire items)	How confident are you that you could eat healthy foods when you are (1) at the shops; (2) hungry after school or work; (3) with your friends; (4) feeling down, bored or stressed; (5) eating out; or (6) alone	Not at all; slightly; moderately; very; extremely

### Diet quality in early adulthood (outcome)

Diet quality in early adulthood, assessed using an adaptation of the Australian Dietary Guideline Index (DGI), was measured at baseline (T1) and follow-up (T3). Diet quality at T3 was used as the outcome variable in the present analysis. While we acknowledge that diet is not a simple behavioural choice but also a result of what is structurally possible for individuals, [32] we have used the term behaviour because it is used within the COM-B framework.

The DGI is a food-based score designed to reflect the diet quality of adults based on adherence to the 2013 Australian Dietary Guidelines [7, 33–36]. The DGI was adapted for use in this study by excluding items for food variety, milk intake, unsaturated fat, and alcohol due to lack of questionnaire items for these indicators. Nine food components were included: (1) vegetables; (2) fruit; (3) whole grains; (4) lean meat; (5) water; (6) foods containing saturated fat, added salt, added sugars and alcohol; (7) foods high in saturated fat; (8) foods and drinks containing added salt; (9) foods and drinks containing added sugars (Supplementary Table 2) [37]. Participants self-reported the number of servings of fruit (excluding 100% fruit juice) and servings of vegetables they usually ate per day using previously validated questionnaire items [37, 38]. Bread type was used as an indicator of grain foods consumed, with seven response options (Supplementary Table 2) [38]. To assess intake of lean meats and alternatives, two indicators of red meat (frequency/week) and fish intake (frequency/week) were available. To assess water intake, two indicators were used: total beverage intake (serves/day of water, fruit juice, diet soft drink, regular soft drinks, cordial and sports drinks) and the proportion of water to total beverages consumed (%).Consistent with definitions of energy-dense, nutrient-poor discretionary foods in the Australian Dietary Guidelines, [33] foods high in saturated fat were represented by five items, [37] including the frequency per week that participants reported they usually ate: ice cream, icy poles and ice blocks; hot chips, wedges and fried potatoes; potato crisps and other salty snacks; confectionery such as lollies and chocolates; and sweet biscuits, cakes and muffins. Frequencies were converted to daily serve equivalents (never = 0;<1/week = 0.07/day;1–2/week = 0.21/day;3–4/week = 0.5/day;5–6/week = 0.79/day). To assess limiting added salt, use of salt during and after cooking was used (never or rarely; sometimes; usually). To assess consumption of foods and beverages containing added sugar, the number of serves/day of soft drink, ice cream/ icy poles, confectionary and sweet foods was determined by combining daily serve equivalents of each item, as described above for saturated fat [37].

The eight individual DGI components were scored out of 10 (zero indicating the guideline was not met) and summed. Items with two components (lean meat, water) were scored out of five. Grains and foods high in saturated fat were also scored out of five as these included two components in the original DGI but information on total cereal and trimmed meat intake were not available in this study. Cut-offs used to obtain the maximum score for each component were tailored to age- and sex-specific food-based recommendations outlined in the Australian Dietary Guidelines [39]. Proportionate (continuous) scores were derived for vegetable and water intake where intakes fell between the maximum and minimum scoring criteria [40, 41] with discrete scores used for fruit, lean meat and foods containing added salt. For fruit, two discrete scores (2.5 and 5 out of 10) were assigned for intakes that fell between the maximum and minimum score criteria. For lean meat, a discrete score of 5 out of 10 was assigned for intakes of red meat 5–6 times/week. For foods containing added salt, scores of 5 out of 10 were assigned for sometimes using salt during and after cooking (Supplemental Table 2). The total modified DGI scores could range between 0 and 80, with a higher score indicating better diet quality.

#### Sociodemographic characteristics and other health-related behaviours in adolescence

Participants reported sex at recruitment (gender identity was not asked) and age was computed from reported date of birth in the baseline survey. MET of leisure-time physical activity in a usual week (min/week) was assessed at baseline using the leisure module from the International Physical Activity Questionnaire (IPAQ) using standard MET values for walking (3.3 METs), moderate (4 METs) and vigorous (8 METs) activities [42, 43]. Self-reported height and weight at baseline were used to derive body mass index (BMI) z-scores. BMI z-scores were estimated using the LMS method (Lambda for the skew, Mu for the median, and Sigma for the generalized coefficient of variation) and using age- and sex-specific BMI percentiles based on the World Health Organization’s BMI-for-age cut-offs [44]. Whether participants were still at school and whether participants still lived at home with their parents or relatives was assessed at follow up (T3).

### Statistical analyses

**Fig. 1:**
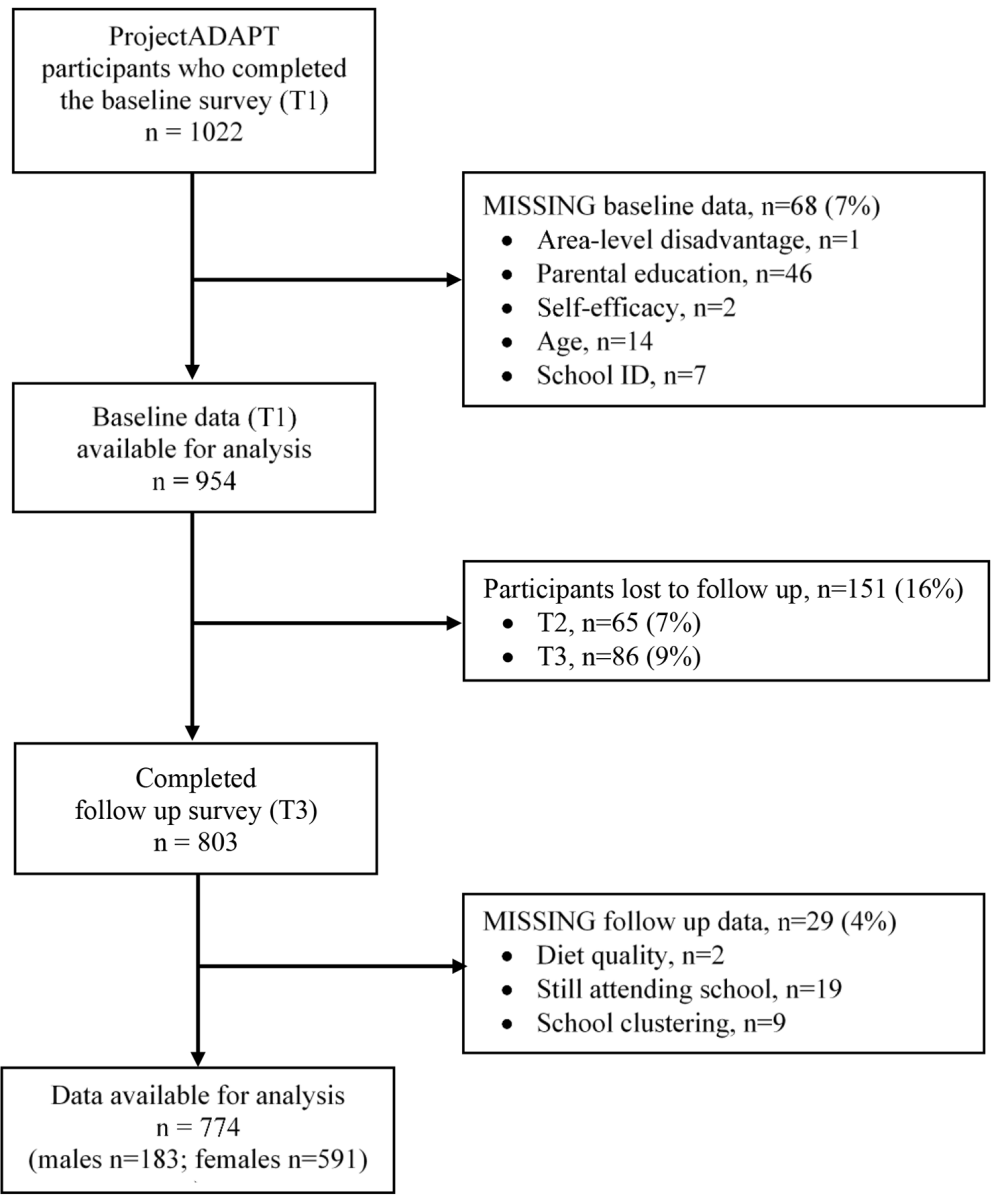
Flow chart of participants included in the analysis of the ProjectADAPT study

The theoretical framework and pathway diagram based on the COM-B model is presented in Fig. 1. Structural equation modelling (SEM) was used to model associations between SEP in adolescence and diet quality in early adulthood and pathways mediated by determinants categorized as Capability, Opportunity and Motivation. SEM is a technique for examining conceptual models that simultaneously assesses all related pathways considering the role of exogenous and endogenous variables [45, 46]. Latent variables were created for Capability, Opportunity and Motivation using the measured variables summarized in Table 1. As illustrated in Fig. 1, SEM was used to test individual indirect effects via associations between SEP and COM (a_1_–a_3_) and associations between COM and diet quality (b_1_–b_3_), controlling for SEP and potential confounders. Confounders included age, sex, baseline diet quality whether still at school and whether living at home with family at follow up. The total indirect effect of SEP on diet quality was assessed, where a*b represents the total individual indirect effect of SEP on diet quality via each COM potential mediator. The direct effect, denoted by c’, is the effect of SEP on diet quality independent of all potential COM mediators and confounders. The proportion mediated was calculated as the mediator-specific indirect effect divided by the total SEP–diet quality effect [a*b/ ((a_1–4_) * (b_1–4_) + c’)], where the total effect represents the sum of the total indirect (indirect effect of all mediators combined) and direct (unmediated) effects. The individual mediating effect of Capability, Opportunity and Motivations on associations between SEP and diet quality were estimated separately, as well as the joint mediating effects (Opportunity -> Capability -> Motivation). Models were fitted using the full information maximum likelihood method and accounting for clustering by schools. Robust clustered (by school) 95% confidence intervals (CI) were estimated for all effects. Models were estimated in the total population and by sex. No statistical interaction was tested by sex due to model non-convergence. Standardized estimates and robust 95% CI are presented. Due to clustering by schools, the fit statistics used to compare models were the Standardized Root Mean Squared Residual (SRMR), with a value < 0.08 indicating a better fit, and the Coefficient of Determination (CD), with a value closer to 1 indicating a better fit [47]. These fit statistics were examined to determine whether the model was a better fit (1) when the latent variable for Opportunity was only based on fruit and vegetable availability at home or also included fruit and vegetable availability in the neighborhood and (2) if confounders related to school attendance and living at home at T3 were included. Descriptive analyses included mean (standard deviation) or median (inter quartile range) for continuous variables and number (%) for categorical variables. Analyses were conducted in Stata using the *sem* and *estat* commands (Version SE 15.0, StataCorp). P < 0.05 was considered statistically significant.

## Results

### Participant characteristics

**Fig. 2:**
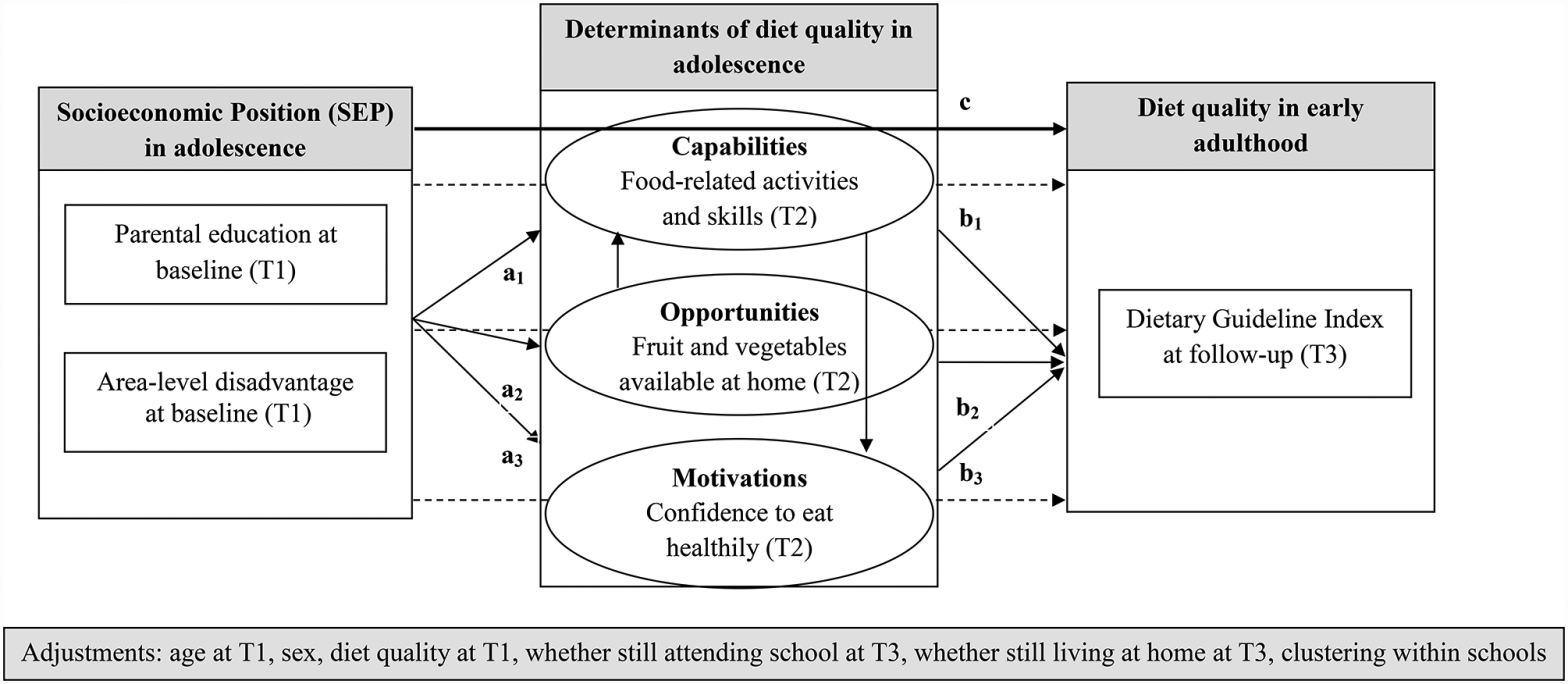
Theoretical framework and pathway diagram for examining the mediating role of adolescent COM in associations between SEP in adolescence and diet quality in early adulthood based on Capability, Opportunity, Motivation and Behaviour (COM-B) model The Endogenous variables (dependent) are diet quality and COM. Parental education and area-level disadvantage are each used as indicators of SEP in separate models. COM represent potential mediators at baseline (latent variables) on the pathway between SEP at baseline (measured variable) and diet quality at T3 (measured variable). In this COM model, Capability was a latent variable designed to represent food-related activities and skills, Opportunity was a latent variable designed to represent availability of fruit and vegetables in the home, and Motivation was a latent variable designed to represent confidence that one can eat healthily (self-efficacy). The indirect mediated effects of SEP on diet quality through Capabilities, Opportunities and Motivations (indirect paths – represented by dashed line) were estimated by multiplying the a-path effects of SEP on each mediator (paths a_1-3_) by the b-path effect of each mediator on diet quality (paths b_1-3_). The individual mediating effect of Capability, Opportunity and Motivations, as well as the joint mediating effects (Opportunity -> Capability -> Motivation) in associations between SEP and diet quality were estimated. The total effect between SEP and diet quality is represented by path c. T, timepoint

Of the 1022 adolescents who completed the baseline survey, 7% (n = 68) of participants were excluded for missing relevant baseline data (Fig. 2). A further 151 (16%) participants were lost to follow up, of which 65 (7%) were lost at T2 and 86 (9%) were lost at T3. Of the 803 participants who completed the T3 follow-up survey, 4% (n = 29) were missing data on diet quality and/or confounders (Fig. 2). A total of 774 participants were included in the present analysis (76% of those who participated at baseline). Mean age at baseline was 16.9 (0.5) years and 76% were female (Table 2). A total of 8%, 26% and 66% of adolescents had parents with a low, medium and high education, respectively. At baseline, mean DGI was 47.9 (13.9) out of a total possible score of 80, while at follow up it was 47.6 (13.6). Only 17% and 18% of adolescents reported making a shopping list and preparing a meal at least twice a week, respectively. Almost all adolescents reported usually or always having fruit (97%) and vegetables (97%) available at home. The proportion of adolescents who were confident they could eat healthily ranged from 16% when feeling down or stressed, to 43% when at the shops. Participant characteristics according to sex, parental education and area-level disadvantage are presented in Table 2.

**Table 2:** Baseline participant characteristics overall and by indicators of socioeconomic position (n = 774)

Characteristic	Overall	Sex		Parental education			Area-level disadvantage		
		Female	Male	Low	Medium	High	High	Moderate	Low
n (%)	774 (100)	591 (76.4)	183 (33.6)	60 (7.8)	198 (25.6)	516 (66.6)	258 (33.4)	258 (33.3)	258 (33.3)
Age, years, Mean (SD)	16.9 (0.5)	16.9 (0.4)	16.9 (0.4)	16.9 (0.5)	16.9 (0.4)	16.9 (0.4)	16.9 (0.5)	16.8 (0.4)	16.9 (0.4)
Female (%)	76.4	-	-	81.7	77.3	75.4	76.4	74.8	77.9
Dietary Guideline Index, Mean (SD)	47.9 (13.9)	48.5 (14.2)	46.1 (13.2)	42.8 (13.6)	46.3 (14.1)	49.1 (13.7)	47.1 (13.9)	46.2 (13.5)	50.4 (14.1)
Leisure time physical activity, MET min/week, Mean (SD)	1852 (2209)	1729 (2110)	2248 (2464)	1701 (2245)	1636 (1995)	1951 (2279)	1991 (2417)	1559 (2068)	2003 (2106)
Overweight or obesity (%)	20.4	20.2	20.7	26.7	26.2	17.4	20.7	24.3	16.0
Capability (> 2 times/week, %)									
Shop for food	25.7	28.4	16.9	31.7	29.3	23.6	29.8	22.1	25.2
Plan meals	32.7	34.7	26.2	31.7	31.3	33.3	32.3	30.2	34.5
Make a shopping list	16.8	18.3	12.0	15.0	14.7	17.8	15.5	19.0	15.9
Prepare a meal	18.4	20.5	11.5	16.7	19.7	18.0	19.4	19.4	16.3
Help to prepare a meal	41.1	44.3	33.3	40.0	41.4	42.1	43.8	43.8	37.6
Opportunity (Usually/always have, %)									
Fruit at home	96.5	96.5	96.7	98.3	92.4	97.9	95.7	96.1	97.7
Vegetables at home	97.3	97.3	97.3	96.7	97.0	97.5	96.9	96.5	98.5
Motivation (Extremely - moderately confident eat healthy foods, %)									
At the shops	43.2	42.5	45.4	31.7	34.9	47.7	38.2	43.4	46.9
Hungry after school or work	31.5	29.4	38.3	26.7	22.7	35.5	26.3	31.4	36.4
With friends	33.2	34.9	27.9	30.0	26.3	36.2	28.7	33.7	37.2
Feeling down or stressed	16.2	13.5	24.6	15.0	8.01	19.4	15.5	13.2	19.8
Eating out	33.5	32.4	33.8	30.0	24.8	37.2	29.5	31.4	39.5
Alone	43.0	42.8	43.7	36.7	33.8	47.3	43.0	40.3	45.7

Area-level disadvantage: High area level disadvantage, 805–993 score; Moderate area-level disadvantage, 993–1053 score; Low area level disadvantage, 1053–1151 score

Parents’ highest education level: Low, some high school or less; Medium, completed high school, or technical or trades school certificate or an apprenticeship; High, university or tertiary qualification. Overweight or obesity was derived using age- and sex- specific body mass index (BMI) percentiles based on the World Health Organization’s BMI-for-age cut offs

### Model fit indices

The SEM model for education had a better fit when the model included confounders for whether adolescents still attended school and lived at home at T3 (SRMR: 0.037 vs. 0.040; CD: 0.435 vs. 0.411). This model was also a better fit when the latent variable for Opportunity was only based on fruit and vegetable availability at home (rather than also including fruit and vegetable availability in the neighborhood) (SRMR: 0.037 vs. 0.040; CD: 0.435 vs. 0.424). Similarly, models for area-level disadvantage had a better fit when confounders related to whether still at school and living at home at T3 were included (SRMR: 0.037 vs. 0.040; CD: 0.435 vs. 0.410) and when the latent variable for Opportunity was based only on fruit and vegetable availability at home (SRMR: 0.037 vs. 0.041; CD: 0.435 vs. 0.437).

### Total effects

There was evidence of a total effect of parental education on diet quality in males (β: 0.121; 95% CI: 0.001 to 0.240), but not overall (β: 0.030; 95% CI: -0.026 to 0.087) or in females (β: 0.001; 95% CI: -0.084 to 0.087). Similarly, there was evidence of a total effect of area-level disadvantage on diet quality in males (β: 0.157; 95% CI: 0.026 to 0.288), but not overall (β: -0.008; 95% CI: -0.074 to 0.058) or in females (β: -0.075; 95% CI: -0.204 to 0.055).

### Direct and indirect effects overall and by sex

**Fig. 3:**
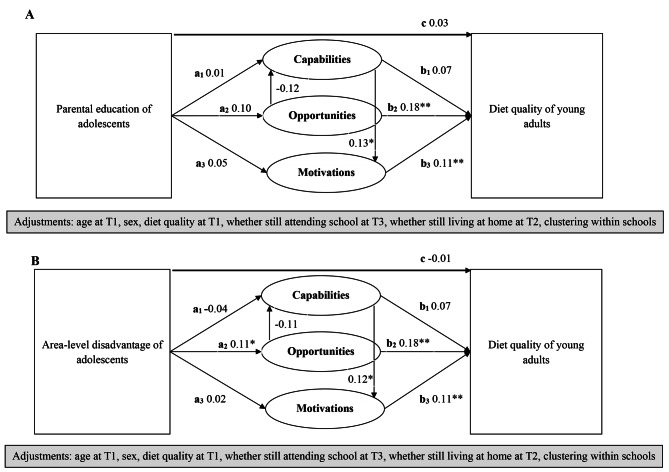
Causal pathway diagram with standardized estimates illustrating the total effects of adolescent SEP on diet quality in early adulthood. Parental education (A), and area-level disadvantage (B), are used as indicators of SEP to examine the mediating role of adolescent dietary determinants in associations between SEP in adolescence and diet quality in early adulthood based on Capability, Opportunity, Motivation and Behaviour (COM-B) model. The endogenous variables (dependent) are diet quality and COM. COM represent potential mediators at baseline (latent variables) on the pathway between SEP at baseline (measured variable) and diet quality at T3 (measured variable). In this COM model, Capability was a latent variable designed to represent food-related activities and skills, Opportunity was a latent variable designed to represent availability of fruit and vegetables in the home, and Motivation was a latent variable designed to represent confidence that can eat healthily (self-efficacy). The mediated effects of SEP on diet quality through Capabilities, Opportunities and Motivations (indirect effect – represented by dashed line) were estimated by multiplying the direct effects of SEP on each mediator (paths a_1 − 3_) by the direct effect of each mediator on diet quality (paths b_1 − 3_). The individual mediating effect of Capability, Opportunity and Motivations, as well as the joint mediating effects (Opportunity -> Capability -> Motivation) on associations between SEP and diet quality were estimated. The association between SEP and diet quality, i.e., total effect, is represented by path c. Significant pathways are indicated as * for p < 0.05 and ** for p < 0.01

For parental education, there was evidence of a direct effect on Capability in males only. While for area-level disadvantage, there was evidence of a direct effect on Opportunity overall and in males only (Table 3; Fig. 3).

**Table 3:** Direct and indirect pathways of association between indicators of socioeconomic position and diet quality mediated by Capabilities, Opportunities and Motivations ^1^

Model path	Direct effects (a-path)^2^ (95% CI)	Indirect effects (a-path x b-path)^3^ (95% CI)	Total effects (c-path)^4^ (95% CI)	Proportion mediated^5^
**Parental education**				
Total population (n = 774)				
Capability	0.007 (-0.083, 0.097)	0.001 (-0.006, 0.007)	0.031 (-0.025, 0.087)	-
Opportunity	0.102 (-0.025, 0.230)	0.018 (-0.003, 0.039)	0.048 (-0.009, 0.105)	-
Motivation	0.047 (-0.033, 0.128)	0.005 (-0.004, 0.014)	0.035 (-0.021, 0.092)	-
Total^6^				
Females (n = 591)				
Capability	0.029 (-0.108, 0.114)	0.002 (-0.007, 0.008)	0.002 (-0.083, 0.086)	-
Opportunity	0.137 (-0.041, 0.316)	0.035 (-0.010, 0.080)	0.037 (-0.031, 0.105)	-
Motivation	0.022 (-0.070, 0.115)	0.002 (-0.010, 0.014)	0.004 (-0.082, 0.090)	-
Total^6^				
Males (n = 183)				
Capability	-0.164 (-0.315, -0.013) *	-0.022 (-0.063, 0.017)	0.098 (-0.025, 0.222)	-
Opportunity	0.046 (-0.084, 0.176)	0.003 (-0.022, 0.042)	0.131 (0.006, 0.256) *	-
Motivation	0.123 (-0.026, 0.291)	0.001 (-0.021, 0.022)	0.121 (0.003, 0.239) *	-
Total^6^				
**Area-level disadvantage**				
Total population (n = 774)				
Capability	-0.043 (-0.132, 0.047)	-0.003 (-0.010, 0.004)	-0.011 (-0.077, 0.054)	-
Opportunity	0.113 (0.023, 0.208) *	0.021 (0.003, 0.038) *	0.012 (-0.055, 0.079)	60.9%
Motivation	0.018 (-0.072, 0.108)	0.002 (-0.008, 0.012)	-0.006 (-0.071, 0.059)	-
Total^6^				
Females^7^ (n = 591)				
Capability	-0.065 (-0.174, 0.044)	-0.003 (-0.011, 0.005)	-0.078 (-0.208, 0.052)	-
Opportunity	0.169 (0.053, 0.284) **	0.046 (-0.040, 0.131)	-0.029 (-0.109, 0.051)	-
Motivation	0.036 (-0.073, 0.146)	0.005 (-0.010, 0.019)	-0.070 (-0.198, 0.059)	-
Total^6^				-
Males (n = 183)				
Capability	-0.031 (-0.195, 0.131)	-0.001 (-0.009, 0.007)	0.156 (0.023, 0.288) *	-
Opportunity	0.012 (-0.130, 0.156)	0.002 (-0.016, 0.020)	0.158 (0.024, 0.293) *	-
Motivation	-0.043 (-0.232, 0.146)	-0.001 (-0.011, 0.009)	0.156 (0.023, 0.288) *	-
Total^6^				

^1^ Standardized coefficients and 95% CI for direct, indirect and total effects were derived using structural equation modelling using the Stata *sem* and *estat* commands; * and ** represent p < 0.05 and p < 0.01 respectively

^2^ The direct effect of SEP on each mediator are represented by paths a_1-3_

^3^ The total indirect effect of SEP on diet quality via each COM potential mediator

^4^ The total effect represents the sum of the total indirect and direct effects

^5^ The proportion mediated was calculated as the mediator-specific indirect effect divided by the total effect [a*b/ ((a1–3) * (b1–3) + c)]

^6^ The total also includes the joint mediating effects of Opportunity -> Capability -> Motivation, which was < 1%

For both SEP indicators, there was no evidence that Capability, which reflected food-related activities and skills, had an indirect effect on diet quality overall or in males and females separately (Table 3; Fig. 3).

For area-level disadvantage, there was evidence that Opportunity, which reflected the home availability of fruit and vegetables, had an indirect effect on diet quality overall. The proportion mediated by Opportunity was 60.9%. There was no evidence of mediation in males and females separately. For education, there was no evidence that Opportunity had an indirect effect on diet quality overall or in males and females separately (Table 3; Fig. 3).

For both SEP indicators, there was no evidence that Motivation, which reflected self-efficacy to eat healthily, had an indirect effect on diet quality overall or in males and females separately (Table 3; Fig. 3).

Across both SEP indicators, there was no evidence of an indirect joint mediating effect by COM (data not shown as coefficients were < 0.001).

## Discussion

This longitudinal study of Australian adolescents used the COM-B model to identify that lower availability of fruit and vegetables at home (Opportunity) explained a large proportion of the association between high area-level disadvantage in adolescence and low diet quality in early adulthood. We found no evidence of mediation by adolescent Capabilities and Motivations, no significant findings for education and no evidence of mediation in males and females separately. These results suggest that strategies to address poor diet quality among adolescents with low SEP should prioritize interventions that target environment-level determinants, rather than individual-level behaviour change.

The present findings for the mediating effect of opportunity on diet quality is consistent with previous research in adolescents with low SEP [48]. In a community-based sample of 2529 adolescents with low maternal education, the home availability of energy-dense snacks and fast foods was a strong mediator of SEP variations in intakes of these foods [48]. Although evidence from mediation analyses in low SEP adolescents is limited, our findings for the importance of home availability of healthy foods is consistent with broader literature on adolescents [14, 49–51]. In the Young Eating Patterns study of adolescents (n = 1,850), home availability of fruits and vegetables was positively associated with change in vegetable consumption 2 years later [14]. In our study, the home availability of fruit and vegetables (Opportunities) explained 61% of the association between neighbourhood disadvantage and diet quality in early adulthood. These findings suggest that dietary interventions and policies should address the availability of healthy foods at home among adolescents living in disadvantaged neighbourhoods. This is particularly relevant for food insecure households, [52] who have been disproportionately affected as a result of the COVID-19 pandemic [53]. In addition, broader social and structural-level factors that influence the home availability of fruits and vegetables should also be addressed, such as ensuring food outlets stock fruit and vegetable varieties that are aligned with cultural beliefs and norms of the communities they serve, increasing the affordability of fruits and vegetables, subsidizing their costs and prioritizing food assistance programs and fiscal incentives for the consumer, food industry and organizations [54–56]. Notably, availability of a large variety of fresh fruit and vegetables in the neighborhood, a further structural-level factor, was excluded from the analyses to optimize model fit.

We found no evidence that food-related activities and skills (Capabilities) and self-efficacy (Motivation) mediated the association between low SEP and low diet quality. A recent COM study [20] found that Motivation (defined as habit strength) was the second most influential factor that explained educational inequalities in fruit and vegetable intake. However, the authors highlight that habits are generated by contextualized-learned behaviours, [57] and that when a habit is established, situational cues in the food environment are important for triggering the behaviour. As a result, although individual motivation may help explain educational inequalities in fruit and vegetable intake in the study in question, there remained a strong focus on environment-level determinants as targets for behaviour change. This narrative was reinforced by Opportunity (financial availability) being the most influential COM mediator identified.

In this study, we only observed evidence of mediation when examining area-level disadvantage, not education. To our knowledge, the two studies to date that have used COM to explain the mediating role of low SEP on diet have only examined one indicator of SEP, i.e. education [20, 50]. Our findings suggests that area-level indicators of SEP may be most relevant for understanding environment-level determinants of diet quality, adding to literature on dietary inequities resulting from living in disadvantaged neighborhoods and experiencing a lack of health promoting resources [49, 58]. Nonetheless, while we refer to education as a measure at the individual level it can reflect inequitable social structures, highlighting the complexity of conceptualizing SEP. Given recent calls for further consideration of the conceptualization of low SEP and construction of categories of social difference, [59] this study adds to the body of literature suggesting that use of different indicators of SEP could identify different priorities for intervention. As COM was a mediator for area-level disadvantage, but not education, in this study, intervention priorities could differ for subgroups who experience disadvantage in different domains.

This study had a number of strengths. The longitudinal design enabled investigation of a period of life where significant life transitions may occur. SEM facilitated examination of the individual and joint mediating effects of multiple food-related determinants of diet quality at multiple levels. A number of limitations should also be acknowledged. The school participation rate was low, and the sample had more females than males, few adolescents had low SEP and fewer adolescents were classified as having overweight or obesity than the national estimates for this age group [60]. Therefore, findings are unlikely to capture the full extent that greater capacity, opportunity and motivation may mediate the relationship between low SEP and diet quality and may not be generalizable to the wider adolescent population. Further, while this study examined findings in males and females separately, the low number of males with low SEP precluded statistical comparisons between males and females. As a result, future research should use targeted recruitment strategies to improve representation of males and adolescents with low SEP. The choice of variables to include in the creation of latent variables was based on mapping of available measures to the COM and model fit statistics, thus not all possible mediators (such as psychosocial capability) could be explored, and some determinants were specific to fruit and vegetables, while others were related to diet quality more broadly. Similarly, this study used parental education and area-level disadvantage as the two SEP indicators, and thus the use of other SEP indicators, such as household income, may yield different findings. Moreover, while Motivation can be defined based on self-efficacy, [9] these are not strictly synonymous, and so future research should consider other indicators from this theoretical domain. As the behavioural and environmental determinants of diet, as well as intervention functions and policy actions, are likely to differ depending on the particular aspects of dietary intake considered (e.g. intake of healthy versus unhealthy foods), future research should examine if findings are consistent when other mediators are examined. Moreover, as most adolescents in this study reported good access to fruit and vegetables at home, future research should confirm whether these findings are consistent in adolescents who report barriers to accessing fruit and vegetables, and greater access to less healthy foods, at home and in their local neighborhood. Lastly, all data were self-reported, which may introduce social desirability and misreporting biases. The systematic bias from dietary misreporting is likely to affect our results. Although non differential error is likely to bias associations towards the null, there is potential for residual confounding due to unmeasured confounders, thus the direction of any biases is challenging to estimate. Furthermore, dietary intake data were collected via a limited number of brief questions, and thus we were unable to capture all elements of the DGI. This limits comparisons with other studies. Lastly, the timeframe of this longitudinal analysis is likely to impact on the generalizability of the findings; young adults were 18–19 years at follow up and therefore unlikely to be fully independent of their home environment (despite controlling for this in the analysis), and further, a longer follow up may have captured greater variation in COM and diet quality, reflective of early adulthood transitions.

## Conclusion

The findings from this longitudinal study of Australian adolescents suggests food-related opportunities at home explain a large proportion of the association between area-level SEP in adolescence and diet quality in early adulthood. These findings highlight that dietary interventions and policies targeting adolescents living in disadvantaged neighbourhoods should prioritize access to fruit and vegetables at home.

## Electronic supplementary material

Below is the link to the electronic supplementary material.

Supplementary Material 1

## Data Availability

The datasets generated and/or analysed during the current study are not publicly available but may be made available by the senior author on reasonable request and upon approval by the Deakin University Human Research Ethics Committee.

## List of Abbreviations

BMI: Body Mass Index
COM-B: Capabilities, Opportunities and Motivations for Behaviour
DGI: Dietary Guideline Index
IPAQ: International Physical Activity Questionnaire
SEM: Structural Equation Modelling
SEP: Socio-economic Position

## References

[1] Australian Institute of Health and Welfare, Poor diet Canberra. AIHW; 2019 [Available from: https://www.aihw.gov.au/reports/food-nutrition/poor-diet.

[2] Australian Institute of Health and Welfare. Nutrition across the life stages. Cat. no. PHE 227.2018.

[3] Australian Institute of Health Welfare (2021). Australia’s youth: Nutrition.

[4] Chong MF-F (2022). Dietary trajectories through the life course: opportunities and challenges. Br J Nutr.

[5] Due P, Krølner R, Rasmussen M, Andersen A, Trab Damsgaard M, Graham H (2011). Pathways and mechanisms in adolescence contribute to adult health inequalities. Scand J Public Health.

[6] Liu J, Rehm CD, Onopa J, Mozaffarian D (2020). Trends in Diet Quality among Youth in the United States, 1999–2016. JAMA.

[7] Livingstone K, Olstad D, Leech R, Ball K, Meertens B, Potter J (2017). Socioeconomic inequities in Diet Quality and Nutrient Intakes among australian adults: findings from a nationally Representative Cross-Sectional Study. Nutrients.

[8] Desbouys L, Méjean C, De Henauw S, Castetbon K (2020). Socio-economic and cultural disparities in diet among adolescents and young adults: a systematic review. Public Health Nutr.

[9] Michie S, van Stralen MM, West R (2011). The behaviour change wheel: a new method for characterising and designing behaviour change interventions. Implement Sci.

[10] Story M, Neumark-Sztainer D, French S (2002). Individual and environmental influences on adolescent eating behaviors. J Am Diet Assoc.

[11] Garcia AL, Reardon R, McDonald M, Vargas-Garcia EJ (2016). Community Interventions to improve cooking skills and their Effects on confidence and eating Behaviour. Curr Nutr Rep.

[12] Morales ME, Berkowitz SA (2016). The relationship between Food Insecurity, dietary patterns, and obesity. Curr Nutr Rep.

[13] Han J, Schwartz AE, Elbel B (2020). Does Proximity to fast food cause childhood obesity? Evidence from Public Housing. Reg Sci Urban Econ.

[14] Pearson N, Ball K, Crawford D (2011). Predictors of changes in adolescents’ consumption of fruits, vegetables and energy-dense snacks. Br J Nutr.

[15] Laska MN, Larson NI, Neumark-Sztainer D, Story M. Does involvement in food preparation track from adolescence to young adulthood and is it associated with better dietary quality? Findings from a 10-year longitudinal study. Public Health Nutr. 2012;15. 10.1017/S1368980011003004 PMC3472035 22124458

[16] Ball K, Lamb KE, Costa C, Cutumisu N, Ellaway A, Kamphuis CBM (2015). Neighbourhood socioeconomic disadvantage and fruit and vegetable consumption: a seven countries comparison. Int J Behav Nutr Phys Activity.

[17] Ziegler AM, Kasprzak CM, Mansouri TH, Gregory AM, Barich RA, Hatzinger LA et al. An ecological perspective of Food Choice and Eating Autonomy among Adolescents. Front Psychol. 2021;12(1098). 10.3389/fpsyg.2021.654139 PMC8097152 33967917

[18] Devine CM (2005). A life course perspective: understanding Food Choices in Time, Social Location, and history. J Nutr Educ Behav.

[19] Willmott TJ, Pang B, Rundle-Thiele S (2021). Capability, opportunity, and motivation: an across contexts empirical examination of the COM-B model. BMC Public Health.

[20] Craveiro D, Marques S, Zvěřinová I, Máca V, Ščasný M, Chiabai A (2021). Explaining inequalities in fruit and vegetable intake in Europe: the role of capabilities, opportunities and motivations. Appetite.

[21] Lachat C, Hawwash D, Ocké MC, Berg C, Forsum E, Hörnell A (2016). Strengthening the reporting of Observational Studies in Epidemiology—Nutritional Epidemiology (STROBE-nut): an extension of the STROBE Statement. PLoS Med.

[22] Fletcher EA, McNaughton SA, Crawford D, Cleland V, Della Gatta J, Hatt J (2018). Associations between sedentary behaviours and dietary intakes among adolescents. Public Health Nutr.

[23] Australian Bureau of Statistics (2013). Socio-Economic indexes for areas (SEIFA) 2011.

[24] Bere E, van Lenthe F, Klepp K-I, Brug J (2008). Why do parents’ education level and income affect the amount of fruits and vegetables adolescents eat?. Eur J Public Health.

[25] Erola J, Jalonen S, Lehti H (2016). Parental education, class and income over early life course and children’s achievement. Res Social Stratification Mobil.

[26] Cane J, O’Connor D, Michie S (2012). Validation of the theoretical domains framework for use in behaviour change and implementation research. Implement Sci.

[27] Berge JM, MacLehose RF, Larson N, Laska M, Neumark-Sztainer D (2016). Family Food Preparation and its Effects on adolescent Dietary Quality and eating patterns. J Adolesc Health.

[28] Boutelle KN, Fulkerson JA, Neumark-Sztainer D, Story M, French SA (2007). Fast food for family meals: relationships with parent and adolescent food intake, home food availability and weight status. Public Health Nutr.

[29] MacFarlane A, Cleland V, Crawford D, Campbell K, Timperio A (2009). Longitudinal examination of the family food environment and weight status among children. Int J Pediatr Obes.

[30] Crawford DA, Ball K, Cleland VJ, Campbell KJ, Timperio AF, Abbott G (2012). Home and neighbourhood correlates of BMI among children living in socioeconomically disadvantaged neighbourhoods. Br J Nutr.

[31] Sallis JF, Pinski RB, Grossman RM, Patterson TL, Nader PR (1988). The development of self-efficacy scales for healthrelated diet and exercise behaviors. Health Educ Res.

[32] Olstad DL, Kirkpatrick SI (2021). Planting seeds of change: reconceptualizing what people eat as eating practices and patterns. Int J Behav Nutr Phys Activity.

[33] Australian Government National Health and Medical Research Council Department of Health and Ageing. Eat for Health. Australian Dietary Guidelines2013 21 October 2016. Available from: https://www.eatforhealth.gov.au/sites/default/files/files/the_guidelines/n55_australian_dietary_guidelines.pdf.

[34] Thorpe M, Milte C, Crawford D, McNaughton S (2016). A revised australian Dietary Guideline Index and its Association with Key Sociodemographic factors, Health Behaviors and Body Mass Index in Peri-Retirement aged adults. Nutrients.

[35] McNaughton S, Ball K, Crawford D, Mishra G (2008). An index of Diet and eating patterns is a valid measure of Diet Quality in an australian Population. J Nutr.

[36] Livingstone K, McNaughton S. Diet quality is associated with obesity and hypertension in australian adults: a cross sectional study. BMC Public Health. 2016;16. 10.1186/s12889-016-3714-5 PMC5045600 27716133

[37] Morley B, Scully M, Niven P, Baur LA, Crawford D, Flood V (2012). Prevalence and socio-demographic distribution of eating, physical activity and sedentary behaviours among australian adolescents. Health Promot J Austr.

[38] Coles-Rutishauser I, Webb K, Abraham L, Allsopp B. R. Evaluation of Short Dietary Questions From the 1995 National Nutrition Survey. 2001.

[39] National Health and Medical Research Council. Educator Guide: National Health and Medical Research Council. Canberra2013.

[40] Waijers PMCM, Feskens EJM, Ocké MC (2007). A critical review of predefined diet quality scores. Br J Nutr.

[41] Wirfält E, Drake I, Wallström P. What do review papers conclude about food and dietary patterns? Food & Nutrition Research. 2013;57. 10.3402/fnr.v57i0.20523. 10.3402/fnr.v57i0.20523 PMC3589439 23467387

[42] Craig CL, Marshall AL, Sjöström M, Bauman AE, Booth ML, Ainsworth BE (2003). International Physical Activity Questionnaire: 12-Country reliability and validity. Med Sci Sports Exerc.

[43] Sjostrom M, Ainsworth BE, Bauman A, Bull FC, Hamilton-Craig CR, Sallis JF, editors. Guidelines for data processing analysis of the International Physical Activity Questionnaire (IPAQ) - Short and long forms2005.

[44] World Health Organization. BMI classification 2015 [updated 21 July 2015. Available from: http://apps.who.int/bmi/index.jsp?introPage=intro_3.html.

[45] Beran TN, Violato C (2010). Structural equation modeling in medical research: a primer. BMC Res Notes.

[46] MacKinnon DP. Introduction to statistical mediation analysis. 1st ed. Routledge; 2008. 10.4324/9780203809556.

[47] Huber C. Introduction to Structural Equation Modeling Using Stata2014. Available from: https://www.stata.com.

[48] Ball K, MacFarlane A, Crawford D, Savige G, Andrianopoulos N, Worsley A (2009). Can social cognitive theory constructs explain socio-economic variations in adolescent eating behaviours? A mediation analysis. Health Educ Res.

[49] McGowan VJ, Buckner S, Mead R, McGill E, Ronzi S, Beyer F (2021). Examining the effectiveness of place-based interventions to improve public health and reduce health inequalities: an umbrella review. BMC Public Health.

[50] Ball K, MacFarlane A, Crawford D, Savige G, Andrianopoulos N, Worsley A (2008). Can social cognitive theory constructs explain socio-economic variations in adolescent eating behaviours? A mediation analysis. Health Educ Res.

[51] Sexton-Dhamu MJ, Livingstone KM, Pendergast FJ, Worsley A, McNaughton SA. Individual, social–environmental and physical–environmental correlates of diet quality in young adults aged 18–30 years. Appetite. 2021:105175. 10.1016/j.appet.2021.105175 33640428

[52] Lindberg R, McNaughton SA, Abbott G, Pollard CM, Yaroch AL, Livingstone KM. The Diet Quality of Food-Insecure australian Adults-A nationally Representative cross-sectional analysis. Nutrients. 2022;14(19). 10.3390/nu14194133 PMC9572719 36235785

[53] Lee AJ, Patay D, Herron L-M, Tan RC, Nicoll E, Fredericks B (2021). Affordability of Heathy, Equitable and more sustainable diets in low-income households in Brisbane before and during the COVID-19 pandemic. Nutrients.

[54] Havdal HH, Fosse E, Gebremariam MK, Lakerveld J, Arah OA, Stronks K (2021). Perceptions of the social and physical environment of adolescents’ dietary behaviour in neighbourhoods of different socioeconomic position. Appetite.

[55] Mozaffarian D, Angell SY, Lang T, Rivera JA (2018). Role of government policy in nutrition—barriers to and opportunities for healthier eating. BMJ.

[56] Gillies C, Super S, Te Molder H, de Graaf K, Wagemakers A (2021). Healthy eating strategies for socioeconomically disadvantaged populations: a meta-ethnography. Int J Qual Stud Health Well-being.

[57] Gardner B (2015). A review and analysis of the use of ‘habit’ in understanding, predicting and influencing health-related behaviour. Health Psychol Rev.

[58] Macintyre S (2007). Deprivation amplification revisited; or, is it always true that poorer places have poorer access to resources for healthy diets and physical activity?. Int J Behav Nutr Phys Activity.

[59] Dijkstra I, Horstman K (2021). Known to be unhealthy’: exploring how social epidemiological research constructs the category of low socioeconomic status. Soc Sci Med.

[60] Australian Institute of Health Welfare (2020). Overweight and obesity among australian children and adolescents.

